# Mr. Fosbrooke, in Reply to Dr. Pearson

**Published:** 1800-03

**Authors:** T. D. Fosbrooke

**Affiliations:** Horsley, Gloucestershire


### *To the Editors of the Medical and Phyſical Journal.*

GENTLEMEN,

IN the London Medical Review of laſt month, I find a paper of mine is inſerted, in reply to a preceding letter of Dr. Pearſon's, on the ſubject of "Eruptions appearing in the Vaccine Inoculation. Dr. P. objected to the hypotheſis of variolous contamination being the cauſe of the appearance of theſe eruptions; and the object of the paper I ſent, was to maintain the very point which Dr. P. reprobated. The *datum* I aſſumed (I preſume a very reaſonable one) was, that "*Eruptions do not appear in the practice of this neighbourhood*;" and that, therefore, till Dr. P. could aſſign a ſatisfactory cauſe for this circumſtance, there would ſtill be a heſitation, indeed an abſolute refuſal, to admit that the vaccine poiſon does, *per ſe*, produce the variolous-like eruptions in queſtion.

Since the days of Lord Bacon, argument has not been allowed in Natural Philoſophy further than in the way of deduction from fact or experiment; and to this rule, this law of modern philoſophizing, I preſume, the further arguments I ſhall now proceed to deduce, in reply to Dr. P. will not be found oppoſite.

Theſe eruptions do certainly not proceed (as a large burden in ſevere ſmall-pox) from peculiar virulence in the diſeaſe; for in a ſtate of the moſt virulent prevalence, (I ſpeak poſitively from immediate obſervation) cow-pox has not been found to vary its uſual aſpect.

If, then, theſe eruptions do not conſecutively follow virulence in the diſeaſe, they do not proceed from the diſeaſe itſelf, but from the conſtitution of the patient. There can be no other reaſon aſſigned, why in two perſons inoculated with the *ſame matter*, one ſhall have the diſeaſe worſe than the other.

other. What then eſtabliſhes virulence in this diſeaſe (excluding accidents and circumſtances) but conſtitution? Of courſe, virulence and conſtitution are ſynonymous, and eruptions cannot be the natural and neceſſary conſequence of one or the other.

If, then, theſe eruptions are not the effect of virulence or conſtitution, from whence either do they or can they proceed, but from the ſuppoſition of variolous contamination?

I underſtand, that cow-pox is not communicable by effluvia, *without* theſe eruptions; how comes it to have it *with* them? (In fact, any change of property acquired through eruptions, has the ſame force in the argument as a datum). If this contagious property, or rather variolous, appertained to pure cow-pox, it ought to have it in one ſtage as well as another. This is a dilemma which, I preſume, nothing but the hypotheſis of variolous contamination can reſolve.

It ſeems to me an exception to the uſual claſſifications of the Noſologiæ, that the ſame diſeaſe ſhould poſſeſs contrary properties, or, indeed, that any thing elſe ſhould, at the ſame time. In pure phyſics, aſſuredly, it cannot: yet here is cow-pox pure in one patient, and variolated cow-pox in another. Analogous cauſes produce anomalous effects.

Theſe eruptions are not ſecondary, as in cow-pox; but primary, as in ſmall-pox.

Inoculated cow-pox is plainly a topical diſeaſe, as to outward aſpect; how comes it that it ſhould loſe this locality of aſpect, and become like ſmall-pox?

In ſmall-pox, ſomething is expelled by Nature externally from the ſkin, viz. puſtules; but cow pox has the ſame effect, without operating in this mode upon the ſkin, by alteration without expulſion. If ſo, with ſimilar conſequences, the two diſeaſes are diſſimilar in operation; how come they, then, to be ſimilar?

Small-pox certainly has ſometimes a ſimilar effect, but this is plainly owing to its mildneſs; and in a virulent ſtate, it is always attended with a heavy burden: but this, it has been proved, is not a conſequence of virulence in cow-pox, (however Dr. Woodville's claſſical and elegant pamphlet would inſinuate as much*) and therefore the argument from hence fails. But

* If Dr. W. could pronounce Cow-pox, with all his bad caſes of it, a much milder diſeaſe than Small-pox, what muſt it be in the practice of it elſewhere? I allow Dr. Woodville all poſſible commendation for his pamphlet; but I conſider this, that were it not for the anomalous aſpects it exhibits, it contains nothing which was not known before, and therefore, *without*

But why may not cow-pox be ſimilar to ſmall-pox? Why may it not produce eruptions? It cannot be ſo; becauſe, if it did, it would at all times have ſimilar properties; *e. g.* that of communicating infection by effluvia, which it has not. At leaſt, it would be ridiculous to infer, that it *acquires* this property—by what? by *appearing* in the form of eruptions! I do not proceed with this *reductio ad absurdum.*

I have mentioned virulence in cow-pox. Owing to the neglect of advice, my own child had it exceedingly ſevere. All the conſequence, however, was, not *contagion by effluvia*, not *variolous eruptions*, not *convulſions*, not one *Woodvilleian* appearance, but laſſitude, the gradual decay of old puſtules, and the emiſſion of new ones, around the inoculated part. All this, which, I underſtand by Dr. Jenner's publications, is ſecondary diſeaſe, was plainly local; and was cured by me in leſs than a week, by a few drops of diluted vitriolic acid, applied with the end of a feather upon the puſtules, in the rudeſt manner. I am,

GENTLEMEN,

Your moſt obedient ſervant,

T. D. FOSBROOKE, M. A. F. A. S.

*without* thoſe anomalous aſpects, would not be worthy of publication. And after all, the whole of its credit is equivocal. The public will at leaſt concede, that if with experience equally extenſive, we ſtill find the reſults the ſame as originally ſtated, we have no right to reſign thoſe original pretenſions.

